# XBB1.5-Adapted COVID-19 Vaccine Acceptance Among Dialysis and Kidney Transplant Patients: A Bi-National Survey Study

**DOI:** 10.3390/vaccines13030213

**Published:** 2025-02-21

**Authors:** Georg Beilhack, Rossella Monteforte, Florian Frommlet, Alicia Faltum, Timna Agur, Ruth Rahamimov

**Affiliations:** 1 Division of Nephrology and Dialysis, Department of Medicine III, Medical University of Vienna, 1090 Vienna, Austria; 2 Center for Medical Data Science, Medical University of Vienna, 1090 Vienna, Austria; 3 Faculty of Medical and Health Sciences, Tel Aviv University, Tel Aviv 6997801, Israel; 4 Department of Nephrology and Hypertension, Rabin Medical Center, Petah-Tikva 4941492, Israel; 5 Department of Transplantation, Rabin Medical Center, Petah-Tikva 4941492, Israel

**Keywords:** dialysis, kidney transplant, COVID-19, XBB1.5-adapted vaccine, vaccine acceptance, vaccine hesitancy, booster

## Abstract

**Background**: A decrease in governmental vaccination initiatives and diminishing public enthusiasm for vaccines could jeopardize vaccine uptake, potentially endangering those who are most at risk. In this survey study, we evaluated the current acceptance rates of the newly developed monovalent XBB1.5-adapted COVID-19 vaccine among kidney transplant recipients and dialysis patients in Austria and Israel and identified factors influencing vaccine acceptance. **Methods**: The survey involved a total of 656 patients aged 18 and older and was carried out from 20 November to 21 December 2023, at the Medical University of Vienna, Austria and the Rabin Medical Center in Petah Tikva, Israel. Logistic regression analysis was used to explore the relationships between vaccine acceptance and variables such as age, gender, country, past COVID-19 infection status and severity, renal replacement therapy, education level, and willingness to receive the annual flu vaccine. **Results**: The survey showed that 54% of patients in Austria and 63% in Israel expressed acceptance of the modified XBB1.5-adapted COVID-19 vaccine. The main hesitancy was due to concerns about potential side effects, with 44% in Austria and 53% in Israel expressing apprehension. A willingness to receive the influenza vaccine, older age in Austria, and kidney transplant status in Israel were key predictors of greater COVID-19 vaccine acceptance. **Conclusions**: This study showed that more than 50% of our kidney transplant recipients and dialysis patients were willing to receive the adapted COVID-19 vaccine. Yet, vaccine hesitancy remained a significant barrier even among these high-risk groups, despite the availability of an updated COVID-19 vaccine targeting the Omicron subvariant XBB1.5.

## 1. Introduction

Three years after the development of COVID-19 vaccines against SARS-CoV-2 and amidst recurring surges of new variants, it is crucial to maintain awareness of potential risks associated with COVID-19 infection and uphold vaccination rates.

Global attitudes toward COVID-19 vaccination vary considerably across countries, shaped by differences in culture, religion, trust in healthcare systems, and socioeconomic status [1,2,3]. Austria and Israel, despite having similar population sizes (approximately 9 million), demonstrated markedly different attitudes toward COVID-19 vaccination. At the pandemic’s onset, Austria had one of the lowest vaccination rates in Western Europe [4], whereas Israel emerged as a global leader in COVID-19 vaccination efforts [5,6]. By fall 2023, approximately 70% of the populations in both countries had completed their initial vaccination protocols [7]. However, as in other countries like the USA and Canada, booster uptake has stagnated [8]. This stagnation is likely attributable to the decline in governmental vaccination campaigns and waning public interest which could further undermine vaccine acceptance and pose significant risks to vulnerable individuals [9].

At the time of the current study (20 November to 21 December 2023), only 5% of the Austrian population had received the new COVID-19 vaccine [10], although wastewater monitoring in Austria suggested that the highest incidence of COVID-19 infections was recorded since the pandemic’s onset [11]. In contrast, Israel experienced significantly lower COVID-19 infection rates [12], likely as a result of the war in Gaza, which caused travel limitations and restrictions in everyday social activities.

The aim of this study was to assess the current rates of COVID-19 vaccination acceptance among kidney transplant recipients and dialysis patients. For this purpose, we conducted a survey in Austria and Israel with 400 kidney transplant recipients and 256 patients on dialysis. We specifically asked them about their willingness to receive the newly XBB1.5-adapted monovalent COVID-19 vaccine.

## 2. Methods

### 2.1. Study Population

This survey was conducted jointly at the Division of Nephrology and Dialysis at the Department of Medicine III, Medical University of Vienna, Austria, and at the Department of Nephrology and Hypertension as well as at the Department of Transplantation, Rabin Medical Center in Petah Tikva, Israel, during the period from 20 November to 21 December 2023. The Austrian site treats around 1300 kidney transplant patients and 190 dialysis patients, while the Israeli site cares for approximately 2000 kidney transplant patients and 200 dialysis patients. No COVID-19-specific public health regulations or restrictions on hospitalization and medical services were applied during the study period in either country. Patients who visited our clinics during this time and fulfilled the specified criteria, including being 18 years of age or older, receiving regular dialysis, or having received a kidney transplant, were invited to participate. Patients with a language barrier or a psychiatric disease were excluded from the survey. We approached 694 eligible patients in our centers and asked them if they would like to participate in the survey. A total of 656 patients were included in the study. The study size was limited by the number of patients at our centers and the period of the study. Those who agreed were required to provide informed consent before their participation. This survey aimed to estimate the willingness to be vaccinated with the new monovalent COVID-19 vaccine, which targets the Omicron XBB1.5 virus variant containing XBB1.5-specific sequence changes, relative to the original BNT162b2 backbone. This vaccine (Comirnaty^®^ monovalent COVID-19 XBB1.5 variant-adapted vaccine, Pfizer, New York, NY, USA) was authorized by the European Commission on 1 September 2023. The questionnaire was administered to the patient, and information such as age, gender, education level, dialysis modality, or kidney transplant status was recorded (Supplementary File). Age was determined by subtracting the birthdate from a set date (1 December 2023). For patients who provided only their year of birth, their birthdays were presumed to be on 30 June of that year. The demographic details of the study population are summarized in Table 1.

### 2.2. Statistical Analysis

Descriptive statistics are given as counts and percentages or medians and interquartile ranges, as appropriate. A positive attitude of patients toward the newly adapted COVID-19 vaccine against the Omicron variant XBB1.5 was given if patients answered question 5 with “definitely yes”, “probably yes” or had already received the vaccine (14.3% of participants in Austria and 13.4% in Israel at the time of the survey). The chi-square test was employed to determine associations or dependencies between two categorical variables. Several contingency tables were computed to study associations. Logistic regression analysis was conducted to explore the relationships between the predictor variables (age, gender, country, dialysis or kidney transplantation status, education, previous COVID-19 infection status and severity, willingness to receive the annual flu vaccine) and the dependent variable, willingness to receive the XBB1.5-adapted vaccine. Due to the strong interaction between country and several other factors and due to confounding effects of certain predictor variables with country, we decided to perform separate analyses for the two countries. Logistic regression was performed for each predictor variable separately. For those predictors that were significant in this analysis, a multiple logistic regression model was computed. A *p* value of <0.05 was considered to indicate statistical significance. Statistical analysis was performed with the statistical software R version 4-3.2 for Windows/Mac. We adhered to the STROBE checklist.

## 3. Results

In this survey involving 400 kidney transplant recipients and 256 dialysis patients (total N = 656), the median age was 60.3 years (IQR 49–70), and women accounted for 35% of the population. Among the 656 patients, 69% had already contracted SARS-CoV-2 in the past, of whom 12% required hospitalization, 68% experienced mild symptoms, and 20% showed no symptoms (Table 1).

**Figure 1:**
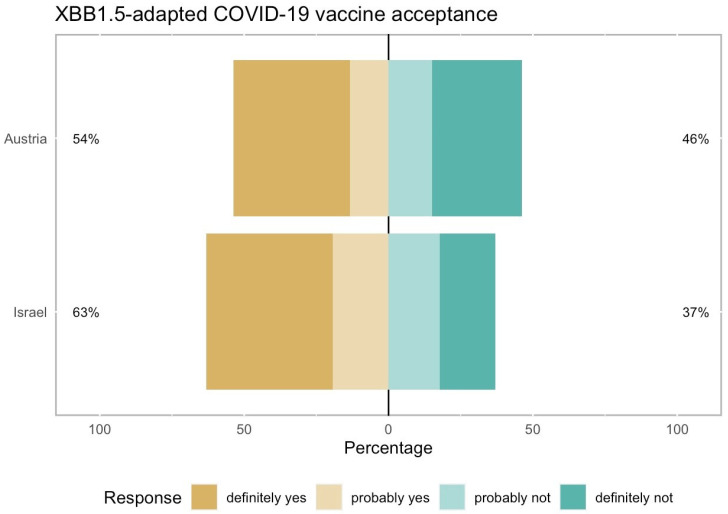
Acceptance rate of the XBB1.5-adapted COVID-19 vaccine among 656 high-risk patients in Austria and Israel. The XBB1.5-adapted COVID-19 vaccine had an acceptance rate of 54% in Austria and 63% in Israel (*p* = 0.015, chi-square test). Patients who had already received the XBB1.5 COVID-19 vaccine were included in the “definitely yes” answer, with percentages of 14.3% in Austria and 13.4% in Israel.

We found that the acceptance rate for the recently modified XBB1.5-adapted COVID-19 vaccine among our patients was 54% in Austria and 63% in Israel (Figure 1), with the rate in Israel being significantly higher than in Austria (*p* = 0.015).

Of these patients, 14.3% in Austria and 13.4% in Israel had already received the modified vaccine. This rate was significantly greater than the general vaccination rate in Austria at the time of the survey, which was 5%. Nevertheless, the current readiness to accept the updated COVID-19 vaccine fell considerably below the pre-2023 vaccination levels in both nations (92%, Table 1).

The primary cause of vaccine hesitancy in both countries was concern over potential side effects or negative long-term consequences, with rates of 44% in Austria and 54% in Israel, respectively (Table 2).

Neither gender, previous COVID-19 infection nor the severity of COVID-19 symptoms affected the willingness of our patients to accept the updated vaccine.

**Figure 2:**
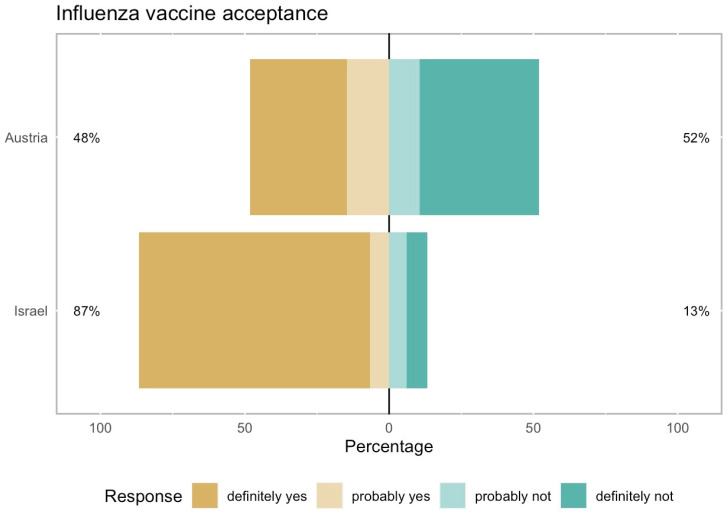
Acceptance rate of the influenza vaccine among 656 high-risk patients in Austria and Israel. The acceptance rate of the annual influenza vaccine was 48% in Austria and 87% in Israel (*p* = 0.0004, Chi-squared test). Patients who had already received the annual influenza vaccine were classified under the “definitely yes” response. Acceptance was defined as providing either a “definitely yes” or “probably yes” answer.

Logistic regression analysis revealed that in both countries, the most significant predictor positively associated with acceptance of the COVID-19 vaccine was willingness to receive the influenza vaccine, with 86.7% of Israeli patients and 48.1% of those in Austria (Figure 2 and Table 3 and Table 4).

The significantly elevated influenza vaccination rate in Israel could be attributed to regular invitations for annual flu shots sent to patients by their healthcare providers.

Additional factors that were positively associated with increased COVID-19 vaccine acceptance included older age in Austria and being a recipient of a kidney transplant in Israel (Table 3 and Table 4). In Israel, the new COVID-19 vaccine was considerably more favored by kidney transplant recipients (70.5%) than by dialysis patients (49.1%) (*p* < 0.0004). An initial univariate logistic regression model indicated a positive association between higher education levels and acceptance rates in Israel (*p* = 0.009). However, in the subsequent multiple regression model, education lost its statistical significance as a predictor of acceptance (Table 3 and Table 4).

## 4. Discussion

Our survey revealed that the majority of dialysis patients and kidney transplant recipients expressed willingness to receive the modified XBB1.5-adapted booster vaccine, despite challenging circumstances such as those in Israel during the ongoing war. The acceptance rate identified in our survey (54% in Austria and 63% in Israel) exceeded our personal expectations, as we noted diminishing interest or even aversion toward COVID-19 booster vaccinations among our patients just prior to conducting the survey. However, the new booster vaccine acceptance rates must be considered as being relatively modest when compared to the vaccination rate of our patients before 2023 (92%).

When the initial anti-SARS-CoV-2 vaccines were released, numerous studies on COVID-19 vaccine acceptance were carried out, demonstrating a substantial acceptance rate among both patients and the general public [13,14,15,16,17,18,19,20,21].

A survey conducted in summer of 2021 found that Austrian adults exhibited relatively high levels of willingness to receive yearly the COVID-19 vaccine (87.1%) [22]. Despite the high initial willingness, vaccine uptake lagged behind the expressed intent. By December 2021 about 68% of the Austrian population completed the initial vaccination protocol [23] and, by August 2022, 59% had received the first booster dose [8]. Vaccine uptake was higher in older adults, while younger, lower-income and rural populations tended to have lower vaccine uptake [24].

In Israel, a survey carried out between December 2020 and May 2021 reported that 83.7% of the general population and 85.7% of chronically ill patients were willing to receive the COVID-19 vaccine [25]. At this time, Israel was one of the global leaders in COVID-19 vaccinations, with 60% of the population with at least one dose of COVID-19 vaccination [7].

A review by Roy et al. identified 58 studies investigating initial COVID-19 booster dose acceptance across various groups, including the general public, healthcare workers, university faculty, and older adults [26]. The review found a global COVID-19 booster acceptance rate of 77.1%, with regional variations: 79.1% in Asian countries, 66.9% in American countries, and 85.4% in European countries. However, concerning data of very low COVID-19 vaccination rates observed during the 2023–24 respiratory virus season are highlighted in a report published by the Center of Disease Control, USA [27]. According to this recent report, only 15.3% of the 8.8 million healthcare workers in acute care hospitals and 10.5% of the 1.8 million healthcare personnel in nursing homes received the COVID-19 booster.

To increase vaccine acceptance, it is essential to understand the underlying reasons for vaccine hesitancy. In our study, the main reason leading to reduced vaccine acceptance in both countries was the fear of potential side effects and long-term negative consequences, as widely described in previous studies [4,19,28,29], highlighting the need to inform patients about the safety and advantages of vaccination.

The strong positive correlation found between the willingness to receive the adapted COVID-19 vaccine and the influenza vaccine is in line with previously published studies [30,31], indicating that annual vaccination campaigns are instrumental in improving vaccine uptake, as observed in Israel for the influenza vaccination. Furthermore, our study showed that higher vaccine acceptance rates were linked to factors such as advanced age in Austria and transplant recipient status in Israel. An association between older age and willingness to receive COVID-19 vaccines has been reported in dialysis patients as well as in the general population [17,30,32]. This link might be attributed to older individuals having a greater risk perception regarding COVID-19 and more trust in medical advice.

The primary limitations of this study are its cross-sectional design and the relatively small number of survey participants, which was influenced by the size of the centers and the exclusion of patients facing language barriers. Additionally, the mixed methods used to administer the questionnaire, either in person or via telephone, may have influenced the nature and consistency of the responses.

## 5. Conclusions

This study is, to the best of our knowledge, the first to explore the willingness of kidney transplant recipients and dialysis patients to receive the monovalent XBB.1.5-adapted COVID-19 vaccine targeting the Omicron variant. Our findings highlight that vaccine hesitancy remains a major barrier to vaccination even among high-risk groups, despite the availability of an updated vaccine targeting a dominant new variant. Given the proven effectiveness of COVID-19 vaccines in reducing hospitalization and mortality, particularly among at-risk individuals [5,33], it is crucial to continue promoting annual COVID-19 booster shots within these vulnerable populations. While strategies to enhance vaccine uptake in both the general population and patient groups have been widely discussed, our study specifically focuses on kidney transplant recipients and dialysis patients, high-risk individuals who were prioritized during the initial vaccine roll-out. In our study, the primary factor contributing to vaccine hesitancy was concerns about potential side effects and long-term consequences. This emphasizes the need for healthcare providers to address safety concerns and build trust by discussing vaccination directly during routine clinical visits.

## Figures and Tables

**Table 1:** Baseline patient characteristics.

	Participants, No. (%)		
Characteristic	All (N = 656)	Austria (N = 350)	Israel (N = 306)
Age, median (IQR)	60.3 (49–70)	58.1 (47–67)	62.6 (50–72)
*Gender*			
Female (%)	232 (35)	122 (35)	110 (36)
Male (%)	424 (65)	228 (65)	196 (64)
*Education*			
Primary/Middle school	251 (41)	225 (64)	26 (10)
Secondary school	188 (31)	72 (21)	116 (44)
College/University	172 (28)	53 (15)	119 (46)
No answer given	45 (7)	0	45 (15)
*Renal replacement therapy*			
Kidney transplant	400	200	200
Dialysis	256	150	106
Vaccinated against COVID-19 prior to 2023	599 (92)	319 (91)	280 (92)
Vaccinated against influenza in the past	499 (77)	222 (63)	277 (91)
*Prior SARS-CoV-2 infection*	450 (69)	264 (75)	187 (61)
No symptoms	88 (20)	38 (15)	50 (27)
Mild symptoms	305 (68)	197 (75)	108 (58)
Hospitalization needed	56 (12)	27 (10)	29 (15)

**Table 2:** Patients rejecting the newly adapted corona vaccine and their justifications.

	Participants, No. (%)		
	All (N = 269)	Austria (N = 160)	Israel (N = 109)
“I am afraid of side effects or long-term negative effects”	130 (48)	71 (44)	59 (54)
“I already had COVID-19”	40 (15)	22 (14)	18 (17)
“I believe that the vaccine does not protect from COVID-19 infection”	62 (23)	42 (26)	20 (18)
“I believe that the Corona virus is not dangerous anymore”	37 (14)	25 (16)	12 (11)

**Table 3:** Univariate logistic regression analysis for acceptance of COVID-19 vaccine (odds ratio with 95% confidence interval).

Characteristic	Austria		Israel	
	OR (95% CI)	*p* Value	OR (95% CI)	*p* Value
Age	1.007 (1.003, 1.010)	0.00015	1.002 (0.998, 1.005)	0.31
Gender (Male)	1.019 (0.913, 1.138)	0.73	1.079 (0.964, 1.208)	0.19
Education	1.039 (0.968, 1.115)	0.29	1.125 (1.030, 1.228)	0.009
Renal replacement therapy (Dialysis)	0.97 (0.873, 1.079)	0.58	0.807 (0.722, 0.902)	0.0002
Influenza vaccine hesitancy	0.859 (0.828, 0.890)	<0.0001	0.8 (0.757, 0.846)	<0.0001

**Table 4:** Multiple logistic regression analysis for acceptance of COVID-19 vaccine (odds ratio with 95% confidence interval).

Characteristic	Austria		Israel	
	OR (95% CI)	*p* Value	OR (95% CI)	*p* Value
Age	1.005 (1.002, 1.009)	0.0016	n.i.m.	n.i.m.
Renal replacement therapy (Dialysis)	n.i.m.	n.i.m.	0.808 (0.723, 0.904)	0.0002
Education	n.i.m.	n.i.m.	1.078 (0.992, 1.172)	0.076
Influenza vaccine hesitancy	0.865 (0.834, 0.897)	<0.0001	0.817 (0.770, 0.866)	<0.0001

n.i.m.: not in model.

## Data Availability

The data sets used and/or analyzed during the current study are available from the corresponding author on reasonable request.

## Supplementary Materials

The following supporting information can be downloaded at: https://www.mdpi.com/article/10.3390/vaccines13030213/s1, Questionnaire.
