# Supplementary material for: XBB1.5-Adapted COVID-19 Vaccine Acceptance Among Dialysis and Kidney Transplant Patients: A Bi-National Survey Study
Source: Vaccines (Basel). 2025 Feb 21;13(3):213. doi: 10.3390/vaccines13030213 (PMC11945776; doi:10.3390/vaccines13030213)
Supplement: Supplementary file 1 [file vaccines-13-00213-s001.zip › vaccines-3483128-supplementary.pdf]

Supplementary Material

XBB1.5-Adapted COVID-19 Vaccine Acceptance Among Dialysis and Kidney Transplant Patients: A Bi-National Survey Study

Georg Beilhack, Rossella Monteforte, Florian Frommlet, Alicia Faltum, Timna Agur and Ruth Rahamimov

Table of Contents:

Questionnaire……………………………………………………………………………………….2-3

<u>Questionnaire</u>

<u>Anti-SARS-CoV-2 booster vaccine acceptance in patients on dialysis and after kidney transplant</u>

Dear Patient,

You are invited to participate in a brief survey regarding the new anti-Corona (Anti-SARS-CoV-2) booster vaccine. This survey is being carried out in our department among patients undergoing dialysis or with a kidney transplant. Your responses will provide valuable insights into your concerns and willingness to receive the vaccine. Your personal details will remain confidential. Your answers will have no impact on your dialysis or medical treatment.

Date:

Patient ID: _____________

<u>Demographics</u>

Year of Birth:

Gender: ◯ Female ◯ Male ◯ Diverse

Dialysis Modality/Kidney Transplant: ◯ HD ◯ PD ◯ Kidney Transplant

Level of education: ◯ Up to 9th grade ◯ High school graduate ◯ College/university graduate

---

<u>Patient survey</u>

1. **<u>Have you been vaccinated for COVID-19 during the pandemic (before 2023)?</u>**

◯ Yes
◯ No

2. **<u>Have you had a corona virus infection?</u>**

◯ Yes
◯ No
◯ I am not sure

3. **<u>If you were infected, were you:</u>**

◯ without symptoms
◯ with symptoms and not hospitalized (cough, fever, fatigue, muscle aches)
◯ hospitalized because of COVID-19

4. **An adapted booster vaccine against the new Corona Virus variant is now available. Did you get it already?**

◯ Yes
◯ No

5. **If you answered the last question with "No", will you get the vaccine if it is recommended by your doctor?**

◯ Definitely yes
◯ Probably yes
◯ Probably not
◯ Definitely not

6. **If you answered question 5 with „Definitely not"or „Probably not", what is the *main* reason?**

◯ I am afraid of side effects or long-term negative effects
◯ I already had COVID-19
◯ I believe that the vaccine does not protect from COVID-19 infection
◯ I believe that the Corona virus is not dangerous anymore

7. **Have you ever gotten a flu shot?**

◯ Yes
◯ No
◯ I do not remember

8. **Do you plan on getting the flu shot this year?**

◯ Definitely yes
◯ Probably yes
◯ Probably not
◯ Definitely not

Thank you for your participation!
